# Comparison of condylar position in normal occlusion, Class II Division 1, Class II Division 2 and Class III malocclusions using CBCT imaging

**DOI:** 10.4317/jced.58970

**Published:** 2021-12-01

**Authors:** Pilar Rivero-Millán, Jose-Maria Barrera-Mora, Eduardo Espinar-Escalona, Carlos-Antonio González-del Pino, Domingo Martín-Salvador, Jose-Maria Llamas-Carreras

**Affiliations:** 1 Dental Surgeon (DDS). University of Sevilla; 2 Doctoral Degree (PhD). Assistant Professor. University of Sevilla; 3 Doctoral Degree (PhD). Titular Professor. University of Sevilla; 4 Expert in statistics applied to health sciences; 5 Doctoral Degree (PhD). Domingo Martin Salvador. Visiting Professor. The Complutense University of Madrid; 6 Doctoral Degree (PhD). Lecturer. University of Seville

## Abstract

**Background:**

The aim of this study was to establish the condylar position in a group of patients with normal occlusion, compared to Class II Div 1, Class II Div 2 and Class III malocclusions using CBCT imaging.

**Material and Methods:**

Retrospective case-control study carried out by analyzing CBCT images of 80 patients. The sample was divided into 4 different groups with 20 patients each (40 TMJ). All patients were positioned using the Frankfurt plane, parallel to the floor and in maximum intercuspation. The control group included asymptomatic patients with normal occlusion (Less than 2mm of tooth size-arch length discrepancy, positive or negative, 0-2mm overjet, 2-4mm overbite, less than 15o rotations, without facial asymmetries, no previous orthodontic or occlusal treatment, without muscular or articular signs or symptoms in both TMJs) and the experimental group with (class II/1, II/2 and III) malocclusions.

**Results:**

The group with normal occlusion had the condyles centrally positioned within the glenoid fossa. The values obtained in this group were considered as optimal and when compared with the other groups with malocclusions. The results established that the position of the condyle was more posterior in class II/2 and more superior in class III patients than the asymptomatic normal occlusion group.

**Conclusions:**

The data obtained in the asymptomatic group with normal occlusion could be used as a reference for future studies. The comparison of these values with those obtained from analyzing the different sagittal malocclusions show significant differences that could be valuable when establishing the diagnosis and the objectives of the treatment plan in orthodontics.

** Key words:**Condylar position, CBCT, dental malocclusion and condylar concentricity.

## Introduction

The role of the condylar position in the correct functioning of the stomatognathic system has been the center of study and controversy throughout the history of dentistry.

The published literature includes several articles that focus on determining if the condylar concentricity could be the optimum position and whether an eccentricity could be a determining factor in the development of temporomandibular joint (TMJ) disorders.

The scientific discussion is inevitably linked to the use of different diagnostic techniques. In the past, the limitations of the diagnostic techniques that were available made it difficult to study the TMJ, as they only allowed the use of two-dimensional images with a large radiation dose.

The radiographic techniques used to study the TMJ improved greatly thanks to researchers such as Pordes (1), Updegrave (2), Grewcock or Lindblom (3). The development of the cephalometry of Broadbent (4) in 1931, which was later used by several authors such as Gillis (5) or Reisner (6), allowed the measurement of the changes that occur in the mandible in comparison to the rest of the cranium. The use of the laminography by authors such as Brader (7) or Ricketts (8-10), led to improvements in the analysis of the mandibular growth.

The advent of three-dimensional diagnostic techniques allowed us to obtain much more precise images. However, the most precise images were obtained when the cone beam computerized tomography (CBCT) became available, which gave high resolution three dimensional images that allowed the assessment and quantification of the facial osseous tissues in real dimensions (1:1 proportion) with no significant magnification or distortion (11), and therefore giving greater anatomical precision (12). It also implied lower cost, lower radiation dose and lower acquisition time than conventional computerized tomography (CT) (13,14).

Despite magnetic resonance imaging (MRI) being considered the gold standard technique to compare soft tissues and visualize complex movements of the disc in multiple views (15-18), CBTC obviously provides some advantages in comparison. Its greater availability, lower cost and higher precision in demonstrating hard tissue components have made the CBCT an accepTable imaging technique to assess the TMJ. Therefore, it provides unique characteristics to daily orthodontic practice (19).

When reviewing some of the most outstanding studies on this topic, authors such as Farrar and Mc Carty (20,12) or Lindblom (22) linked the eccentricity of the condyle with the presence of TMJ disorders. Similarly, authors such as Rokni (23) or Weinberg (24) suggested that the condylar concentricity was the ideal position.

Pullinger (25) studied asymptomatic subjects and concluded that, despite 50-65% of the condyles being in concentric position, there were also patients without concentric condyles that did not suffer from TMJ disorders.

In 1982, After reviewing the literature, the American Dental Association (ADA) concluded that there was insufficient scientific evidence to relate an eccentricity and the presence of TMJ disorders (26).

However, in the following years several studies, such as Cholasueka *et al*. (27) or Incesu *et al*. (28), linked a posteriorly positioned condyle with joint disorders.

Rodrigues *et al*. (29,30), described how the functional load applied to the TMJ can influence its morphology. This load can vary depending on the dento-facial morphology of the subject. Therefore, it can be suggested that both condyle and mandibular fossa will vary in shape in patients with different malocclusions.

The aim of this study was to further investigate the three dimensional condyle position in the glenoid fossa of patients with normal occlusion in comparison to patients that suffer from different types of malocclusion.

## Material and Methods

A non-experimental cross-sectional study was carried out with case-control methodology, which included 4 groups with 20 patients each (40 joints). The control group included asymptomatic patients with normal occlusion and the other three groups included patients with different malocclusions (class II/1, class II/2 and class III). In total, 80 patients were included in the sample, with a total of 160 TMJ (since both joints were analyzed, right and left). The inclusion and exclusion criteria are shown in  Table 1 and  Table 2.

**Table 1:**
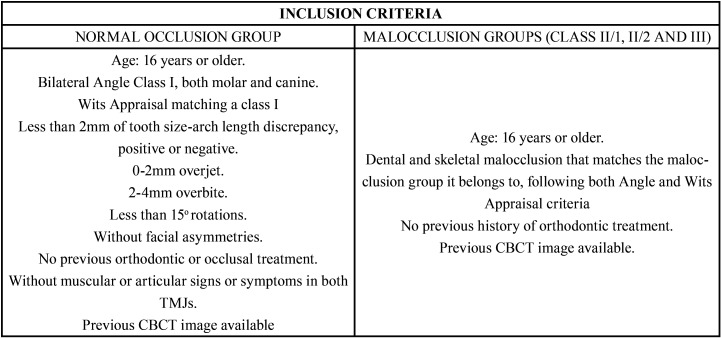
Inclusion criteria.

**Table 2:**
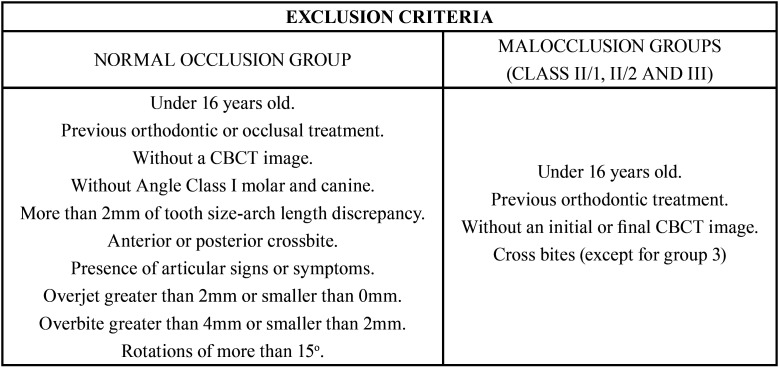
Exclusion criteria.

The program that has been used to perform the measurements is In Vivo Anatomage®, allows obtaining highly accurate quantitative measurements. All CBCTs have been analyzed by the author of the work, previously trained. In addition, to provide internal validity to the measurements taken, we have carried out the intraclass relationship coefficient (ICC). A result of 0.82 was obtained, indicating agreement.

To reach a confidence level of 95% and a statistical power of 80% in the analysis of independent groups, it is necessary a sample of 20 individuals in each group. For intragroup analysis and to achieve the same confidence and power levels, only 12 individuals were required per group.

Hence, we select a sample of 20 individuals per group to cover both scenarios.

CBCT scans were not carried out for the purpose of this study. They were obtained in previous orthodontic or multidisciplinary consultations in a private orthodontic practice. All CBCT scans were performed in the same radiographic center using a Kodak 9500 machine and using the S3D Imaging Software.

All patients were positioned using the Frankfurt plane, parallel to the floor and in maximum intercuspation. All CBCT images were exported as Dicom files and digitalized using In Vivo Dental Anatomage 5 Software, which allows the identification of reference points with great precision and the selection of the view point; it also provides very precise quantitative measurements.

The obtained data was incorporated in an Excel file that was created and codified specifically for the purpose of this study, and that was transformed into a SAV file for statistical interpretation using the software SPSS 24. Statistical hypothesis testing involved an initial normality test of the quantitative variables using the Kolmogorov Smirnov and Shapiro Wilks tests. Since the variables followed a normal distribution, parametric tests were used.

Quantitative variables were compared using the Pearson correlation coefficient for normalized variables.

The mean of variables that were both quantitative and categorical were compared using Student´s T Test if there were only 2 means, or the ANOVA test if there were more than 2 means.

The study sample included 57 women (71.3%) and 23 men (28.7%). The only group that included more male than female patients was the one with class III subjects. The mean age of the sample was 30.15 years with a standard deviation of ±10.93 years.

When analyzing the intraclass correlation coefficient, the intra-observer variability was 0.82, which shows a good level of conformity (31).

**Figure 1:**
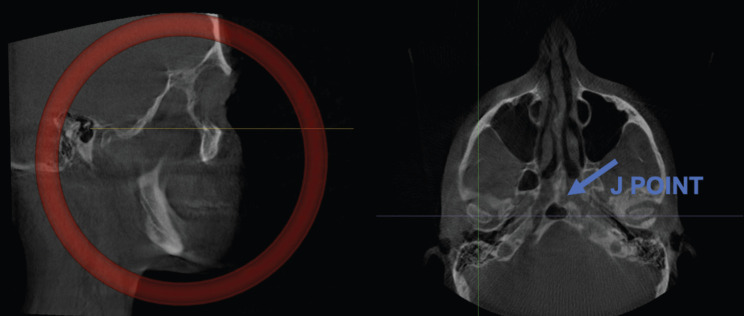
Frankfurt plane parallel to the floor and determination of the J point.

**Figure 2:**
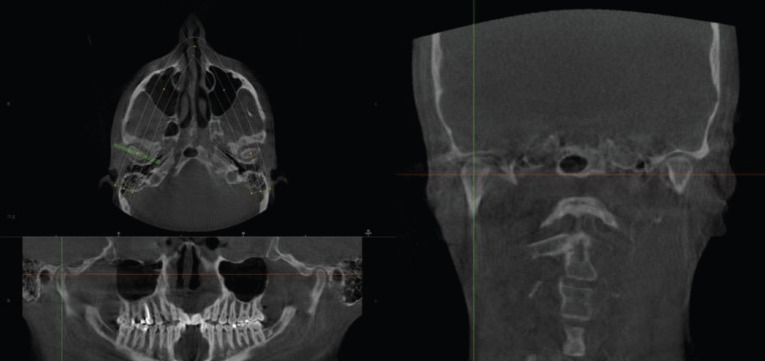
Determination of the midpoint of the condyle.

**Figure 3:**
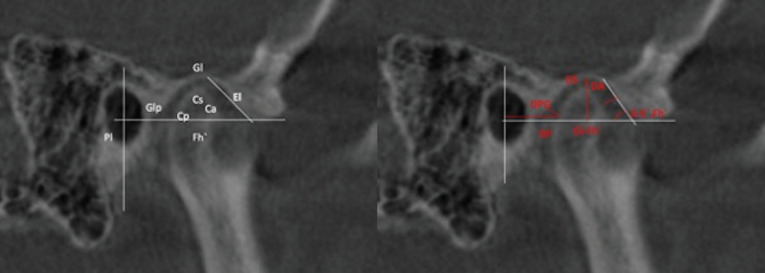
Points, lines and planes analyzed.

**Figure 4:**
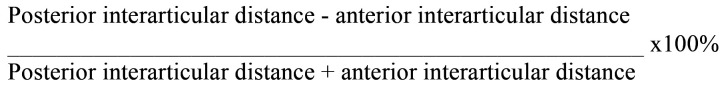
Formula used to obtain the condylar concentricity. Pullinger AG, Hollender L et al.A. A tomographic study of mandibular condyle position in an asymptomatic population. J Prosthet Dent. 1985;53(5):706-13.

All the CBCT images that were analyzed required establishing the Frankfurt plane and reorienting it parallel to the floor. The J point was also determined in the axial plane (union point between the vomer and sphenoid bones) (11), as well as the midpoint of the condyle in both axial and coronal planes (Figs. 1,2). Once the midpoint of the condyle had been established, the sagittal measurements were registered ((Fig. 3,  Table 33, Table 4). Finally, the condylar concentricity was analyzed using the formula described by Pullinger and Hollenger (25), (Fig. 4).

**Table 3:**
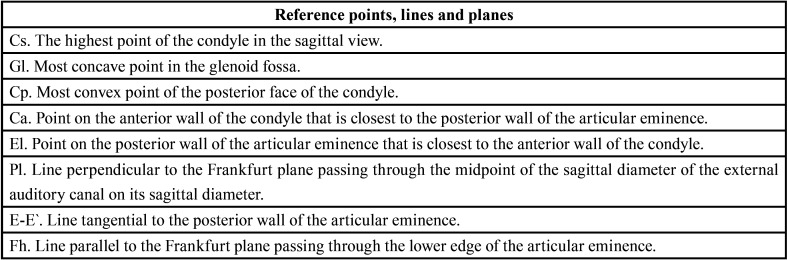
Points and lines analyzed. Taken from de Arieta-Miranda JM et al. Spatial analysis of condyle position according to sagittal skeletal relationship, assessed by cone beam computed tomography. Prog Orthod. 2013;14(1):36.

**Table 4:**
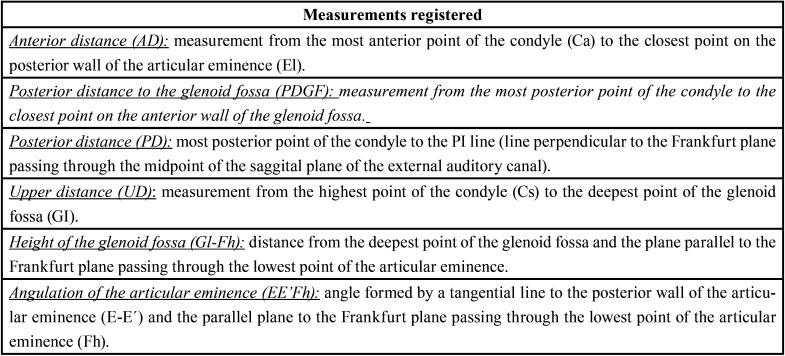
Analyzed measurements. Taken from Arieta-Miranda JM et al. Spatial analysis of condyle position according to sagittal skeletal relationship, assessed by cone beam computed tomography. Prog Orthod. 2013;14(1):36.

## Results

Table 5 shows the results obtained for the variables studied on each group. It should be noted that all the values are given in millimeters except for the angulation of the glenoid cavity (EE’Fh) that was registered in degrees.

**Table 5:**
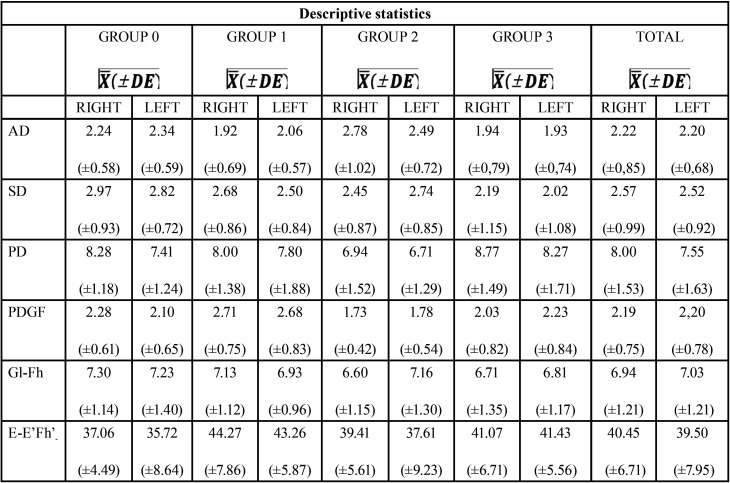
Description of the values and results analyzed.

-Descriptive analysis of the condylar concentricity 

For the purpose of this study, a condyle is considered to have absolute concentricity if the formula described by Pullinger (32) equals to 0. In order to increase the concentricity range, the above author and other authors afterwards, such as Song *et al*. (33) and Krisjane *et al*. (34), established as concentric those condyles that had a value between 0 and 12% using the same formula.

Therefore, a value smaller than -12 reflects a posterior position of the condyle, meanwhile a value greater than +12 indicates an anterior position of the condyle.

In the normal occlusion group, despite that no one in the study sample presented absolute concentricity, 55% had both condyles concentric (values ranging between 0 and ±12), and 12,5% had only one condyle concentric. Therefore, 67.5% or the group with normal occlusion had condylar concentricity in at least one of the two condyles, (Table 6). Only 10% of the sample in groups I (class II/1) and II (class II/2) presented concentricity in both condyles (Table 3). In class II/1 most condyles are positioned anteriorly in the fossa. On the other hand, class II/2 is the group that presented more condyles positioned posteriorly, (Table 7).

**Table 6:**
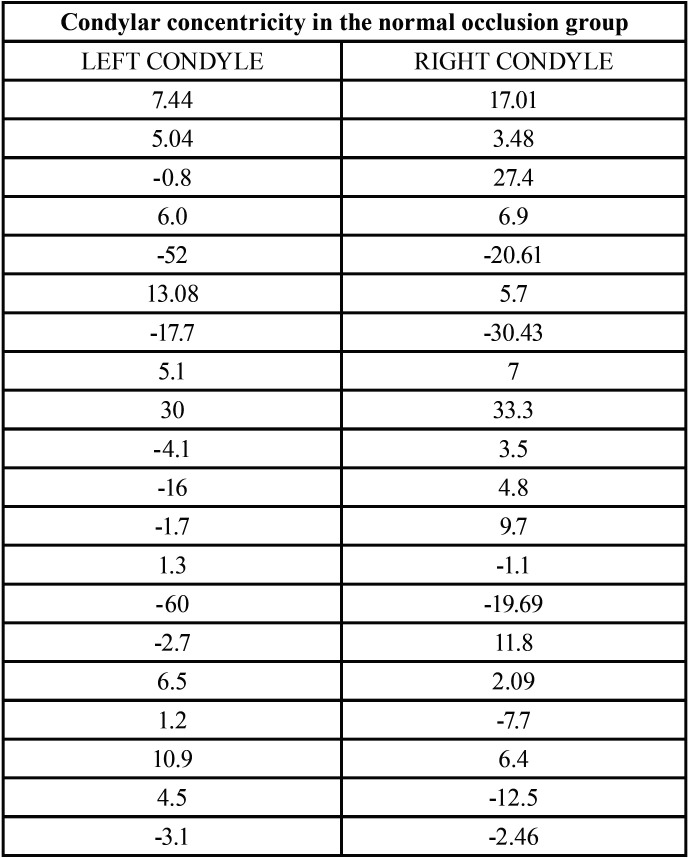
Analysis of the condylar concentricity in the normal occlusion group. The values highlighted in red show that the condyle is concentric.

**Table 7:**
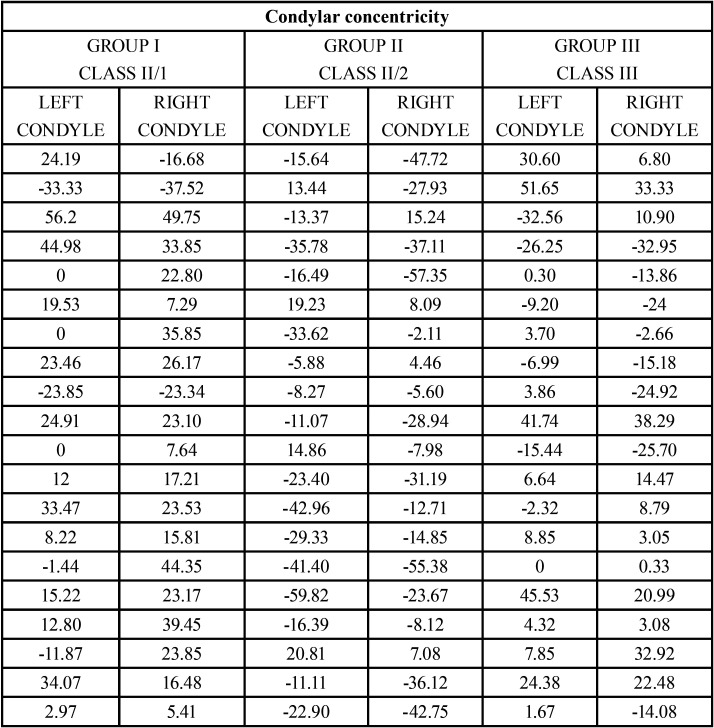
Analysis of the condylar concentricity in the groups with malocclusion.

After analyzing the results, in the control group (normal occlusion) no statistically significant differences were found for both condyles in 5 out of 6 values analyzed. The only measurement that gave statistically significant differences was the Posterior Distance (PD).

To clarify this difference, two different posterior distances where analyzed: the posterior distance (PD) and the posterior distance to the glenoid fossa (PDGF). The *p* value for the posterior distance was 0.029, which is considered statistically significant (*p*<0.05). However, the *p* value for the posterior distance to the glenoid fossa was 2.10 for the left condyle and 2.28 for the right one, and it was established that there was no statistically significant difference between both condyles (*p*>0.05), (Table 8).

**Table 8:**
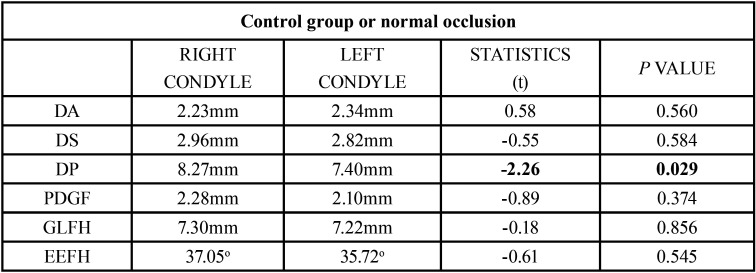
Quantitative analysis of the control group with normal occlusion.

Afterwards, the values obtained for each variable in each malocclusion group were analyzed. Statistically significant differences were found between the different groups when analyzing the Anterior Distance (AD); these differences were on the right condyle, (Table 9).

**Table 9:**
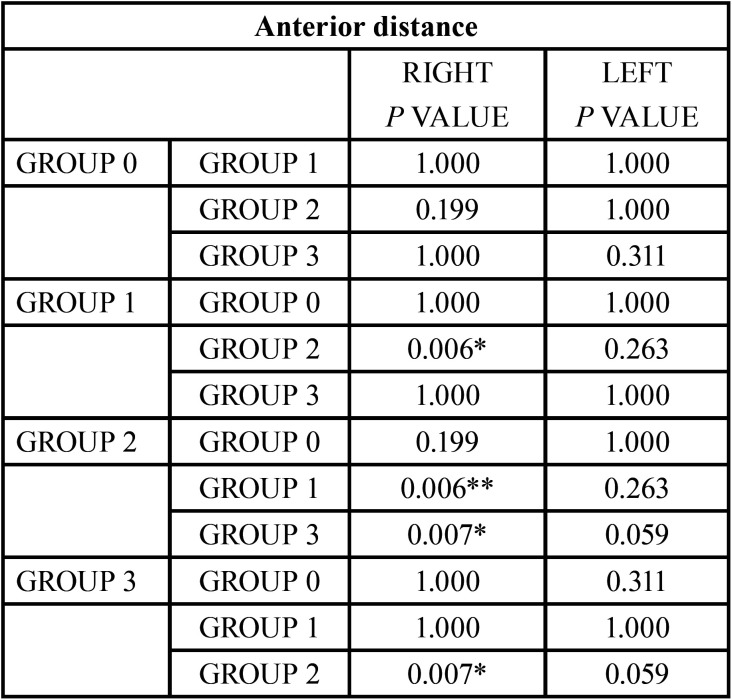
Comparison of the results obtained for each group for the anterior distance. Reference value *p<0.05, **p<0.005.

Like in the normal occlusion group, two different measurements were analyzed to determine the posterior distance of the condile. Statistically significant measurements can be found between some of the groups ( Table 10,  Table 11) in both measurements.

**Table 10:**
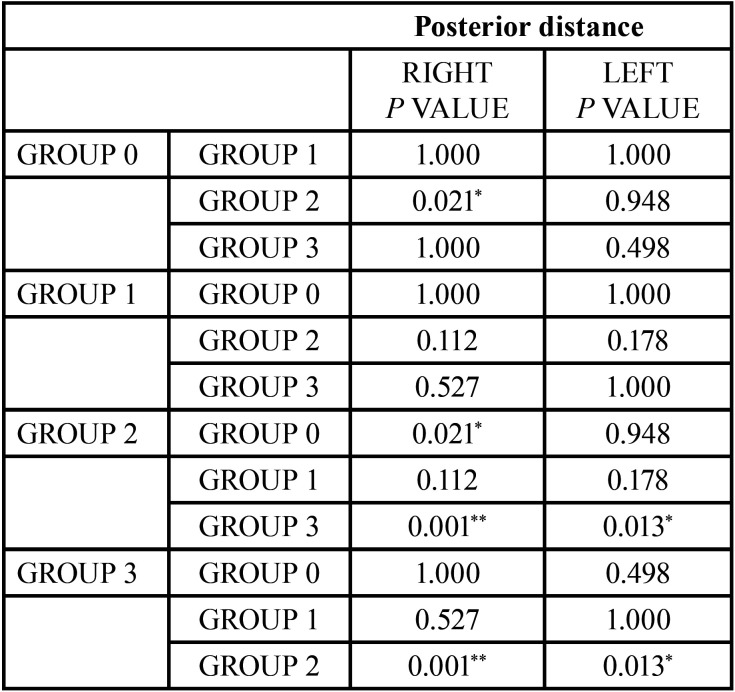
Comparison of the different results obtained for the posterior distance. Reference value *p<0.05, **p<0.005.

**Table 11:**
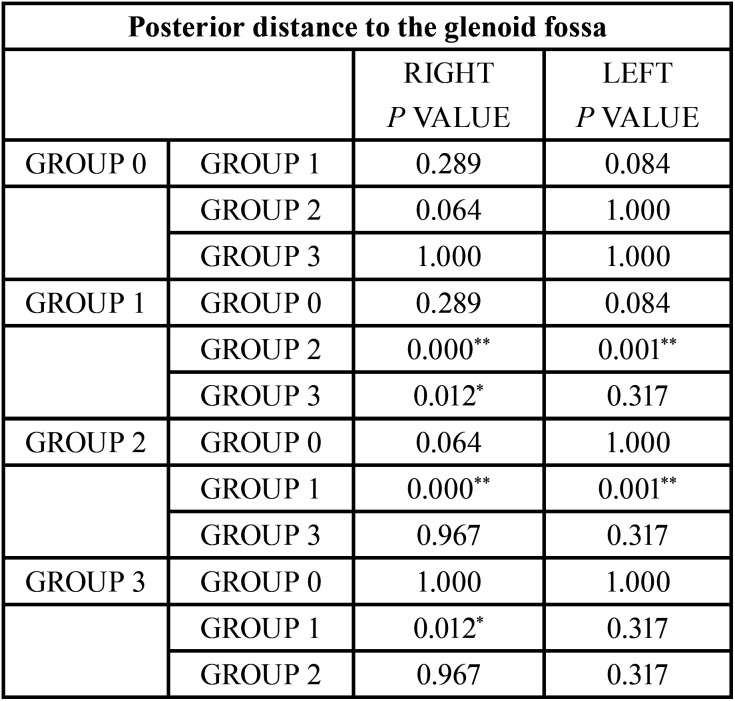
Comparison of the results obtained in the posterior distance to the glenoid fossa. Reference value *p<0.05, **p<0.005.

The analysis of the superior distance and the depth of the fossa are the two measurements that where less statistically significant differences have been found ( Table 12, Table 13).

**Table 12:**
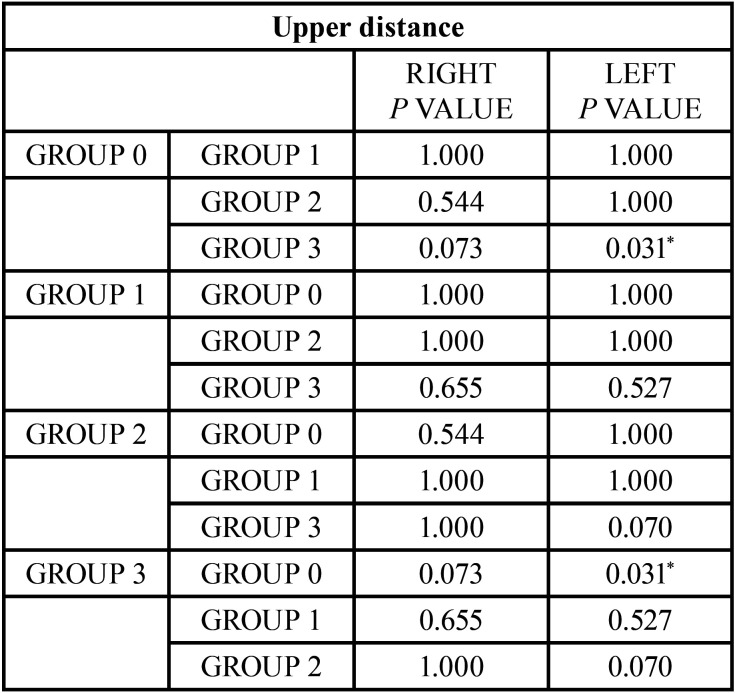
Comparison of the obtained results in the superior distance. Reference value *p<0,05, **p<0,005.

**Table 13:**
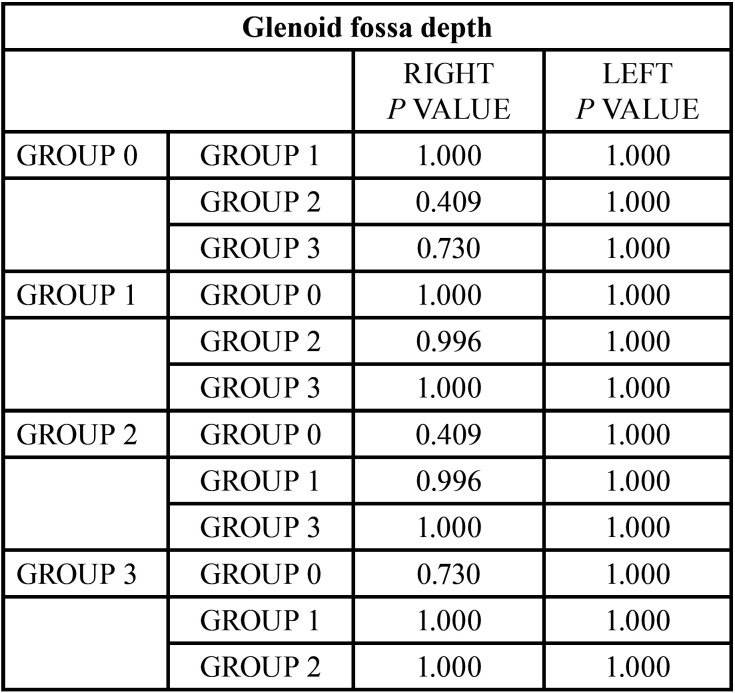
Comparison of the results obtained in the depth of the glenoid fossa. Reference value *p<0.05, **p<0.005

Finally, in the analysis of the angulation of the glenoid fossa ( Table 14) both groups 0 and 1 seem to be statistically significant with the same value for both condyles and on the same when comparing groups 0 and 1.

**Table 14:**
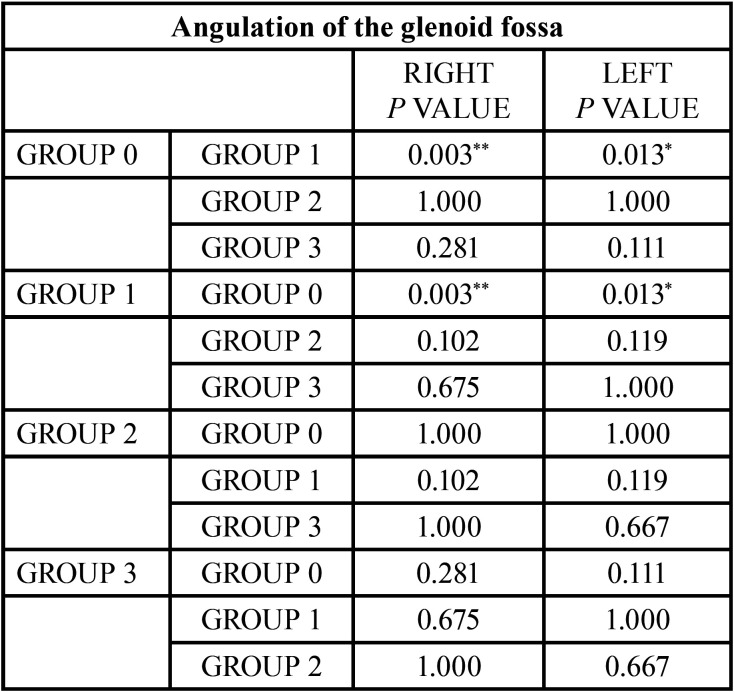
Comparison of the results obtained in the angulation of the glenoid fossa. Reference value *p<0.05, **p<0.005.

## Discussion

The position of the condyle within the glenoid fossa is still controversial, despite the improvements in the diagnostic methods. After reviewing the existing literature, it could be said that many researchers associate the lack of concentricity with internal temporomandibular disorders.

As described by Kurusu *et al*. (35), occlusal forces can modify condylar morphology. Therefore, it is important to know the morphology and the condyle-fossa relationship in the asymptomatic group with an ideal occlusion, as it can be used as a guideline for future diagnosis and treatment planning in patients with malocclusion.

When analyzing the asymptomatic control group with normal occlusion, no significant differences were found between both condyles in five out of six measurements taken; only the posterior distance was significantly different.

As explained in the article written by Miranda *et al*. (11), the posterior distance that uses as reference a perpendicular line to the Frankfurt plane that goes through the midpoint of the external auditory canal, could lead to error due to its morphology and position. To solve this possible error, the current study incorporates the posterior distance to the glenoid fossa.

Five reference measurements were found on this study for which no significant differences were found between both joints. These results match the ones provided by Wang *et al*. (36) when analyzing healthy adults. These values were established as a reference point when analyzing the different sagittal malocclusions.

The greater differences compared to other studies were found when analyzing the depth of the fossa. The results of this study are similar to those described by Miranda *et al*. (11), which is not surprising as it uses the same measuring protocol. However, the results of this study do differ from those of authors such as Vitral *et al*. (37), Song *et al*. (33) or Ganugapanta *et al*. (38), which may be related to the use of a different measuring protocol.

With regards to the condylar concentricity analyzed using the formula described by Pullinger and Hollender, the normal occlusion group is the one with the largest number of concentric condyles. When studying asymptomatic patients (25), these authors described between 50 and 65% of concentric condyles, results that are similar to those obtained in the present study, which were 67.5% concentricity.

Therefore, an association can be made between the high percentage of concentricity of the asymptomatic group with a normal occlusion and a normal function of the TMJ. This theory is supported by authors such as Weinberg (24) y Gerber (35), who have previously linked the condylar concentricity and a normal function of the TMJ.

To determine the spatial relation of the condyle within the glenoid fossa on a sagittal level, the first step was to compare the anterior and posterior distance between the different groups. As exhibited on the results, class II/1 and class III groups have smaller values for the anterior distance, reflecting a more anterior position of the condyle; meanwhile, the class II/2 group has the condyle in a more posterior position. The normal occlusion group displayed intermediate values.

The analysis of the Posterior Distance values provided significant differences between the normal occlusion group and class II/2, and between class II/2 and class III groups. These results agree with those found for the Anterior Distance.

Class III group has an anterior distance similar to Class II/1 group; no statistically significant differences were found, although the posterior distance is greater in class III patients. These results may be linked with the condyle size or the morphology of the glenoid fossa.

These findings back the theory initially described by Thompson (2) in the 80s. He described a posterior displacement in the condylar position in classes II/2 due to a lack of overjet. More recent studies such as Katsavrias y Halazonetis (39) or Zhou *et al*. (40) have observed a more posterior position in the class II/2 group.

On the contrary, authors such as Gianelly and Cohlmia *et al*. (39) found concentric condyles in class II/2 patients. None of these studies could have used CBCT technology due to the date they were carried out on.

Similar results to the ones of this study were found by Song *et al*. (33), where class I, II/1 and III subjects were found to have a mainly anterior and concentric position of the condyle. Class II/2 had a mainly posterior and concentric position of the condyle. Merigue *et al*. (42) and Uzel *et al*. (43) also described a larger number of condyles anteriorly positioned in the class II/1 groups they studied. Krisjane *et al*. (34) also described anterior and concentric positions for class II/1 y III groups.

To complete the sagittal analysis of the condylar position, the obtained values were added using the condylar concentricity analysis formula described by Pullinger and Hollender (34). Similarly, to Cohlmia *et al*. (44), no patients were found to have both condyles in absolute concentricity, but there were multiple condyles within the range considered as concentric.

Class II/1 and class II/2 groups have 27,5% of concentric condyles; this percentage was 45% in class III group.

The percentage of condyles that did not present concentricity seemed to be positioned anteriorly in the glenoid fossa for class II/1 and class III, with this fact being more noticeable in class II/1 group. The group with malocclusion II/2 seemed to have more condyles posteriorly positioned in the fossa.

The Upper Distance was analyzed to determine the condylar position on a vertical level in all groups, and the results were compared. Class III group had higher condyle position within the fossa. However, when compared to the other groups, significant differences were only found compared to the normal occlusion group. These results agree with those of authors such as Kaur *et al*. and Song *et al*. (33), who described a higher condyle position in class III patients.

Katsavrias and Halazonetis (40), which, as previously said, described a more posterior position in class II/2 patients, also established a more anterior position for class II/1 and a higher position in class III. All of their results back the results of this study.

With regards to the morphology of the fossa, its depth and angulation was analyzed following the procedure of Katsavrias and Halazonetis (40). No statistically significant differences were found between any of the studied groups for the studied values to determine the depth of the glenoid fossa. These results do not match those of authors such as Miranda *et al*. (11), Song *et al*. (33) y Katsavrias y Halazonetis (40), as they described a smaller fossa depth in class III patients.

The results obtained when analyzing the angulation of the fossa showed statistically significant differences only between the normal occlusion group and the class II/1 group, with classes II/1 displaying a larger angle.

## Conclusions

1. The normal malocclusion group has a more intermediate position of the condyle within the fossa and a greater percentage of condylar concentricity. The initial hypothesis of this study is supported by this: the optimal position of the condyle would be an intermediate and concentric position within the glenoid fossa.

2. Despite not all of the comparisons between the measurements being statistically significant, they did all support this conclusion: class II/1 and class III malocclusion groups have a more anteriorly positioned condyle compared to class II/2 group, where the condyle is in a more posterior position.

3. Although it was only statistically significant when comparing the normal occlusion and the class III groups: class III patients have the higher condyles position within the glenoid fossa.

4. With regards to the glenoid fossa morphology, no statistically significant differences were found in the depth of the fossa between any of the studied malocclusions. In terms of the angulation of the fossa, Class II/1 presented a greater angle (these results were statistically significant when compared to the normal occlusion control group).
